# Supplementary material for: Enhanced Anxiety and Olfactory Microglial Activation in Early-Stage Familial Alzheimer’s Disease Mouse Model
Source: Biology (Basel). 2022 Jun 20;11(6):938. doi: 10.3390/biology11060938 (PMC9219751; doi:10.3390/biology11060938)

biology

MDPI

Article

Enhanced anxiety and olfactory microglial activation in early-stage familial Alzheimer's disease mouse model

Keerthana Chithanathan[1], Fang-Ling Xuan[1], Miriam Ann Hickey[2] and Li Tian[1*]

Supplementary table S1. Primers designed for *IL1b, IL18, Tnf and GAPDH* genes for expression studies in mice.

Gene name	Primer Sequence 5′-3′	
Il1b	**Forward**	AGCTTCCTTGTGCAAGTGTCT
	**Reverse**	TGGGGTCCGTCAACTTCAAA
Il18	**Forward**	TCAAAGTGCCAGTGAACCCC
	**Reverse**	GGTCACAGCCAGTCCTCTTAC
Tnf	**Forward**	ATGGCCTCCCTCTCATCAGT
	**Reverse**	TTTGCTACGACGTGGGCTAC
Gapdh	**Forward**	GTCATATTTCTCGTGGTTCACACC
	**Reverse**	CTGAGTATGTCGTGGAGTCTACTG

Supplementary figure S1.

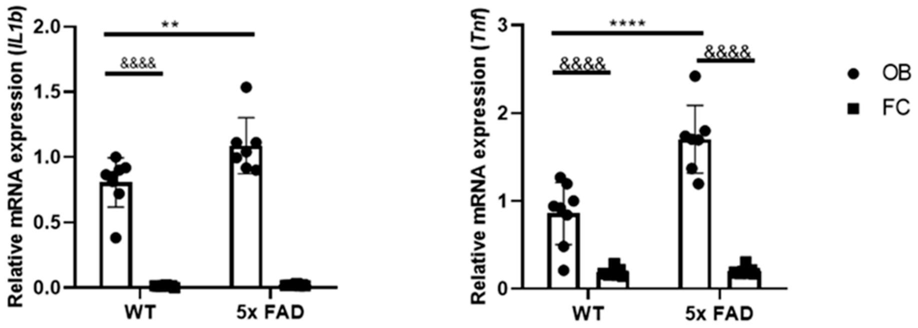

Supplementary figure S1. Higher level of pro-inflammatory cytokines in the OB and their increases in 2-mo 5xFAD male mice. Enhanced gene expression of *Il1b* and *Tnf* as measured by qPCR was seen in the OB compared to the FC; *Il1b* and *Tnf* were also more abundant in the OB of 5xFAD mice than WT littermates (n = 7-8). Data expressed as mean ± SEM; * symbolizes significant genotypic difference while & regional difference; ** $p < 0.01$, &&& $p < 0.001$, **** &&&& $p < 0.0001$ (two-way ANOVA with Tukey's test).

Supplementary figure S2. **Representative dot plots of isotype controls used in flow cytometric analysis**.

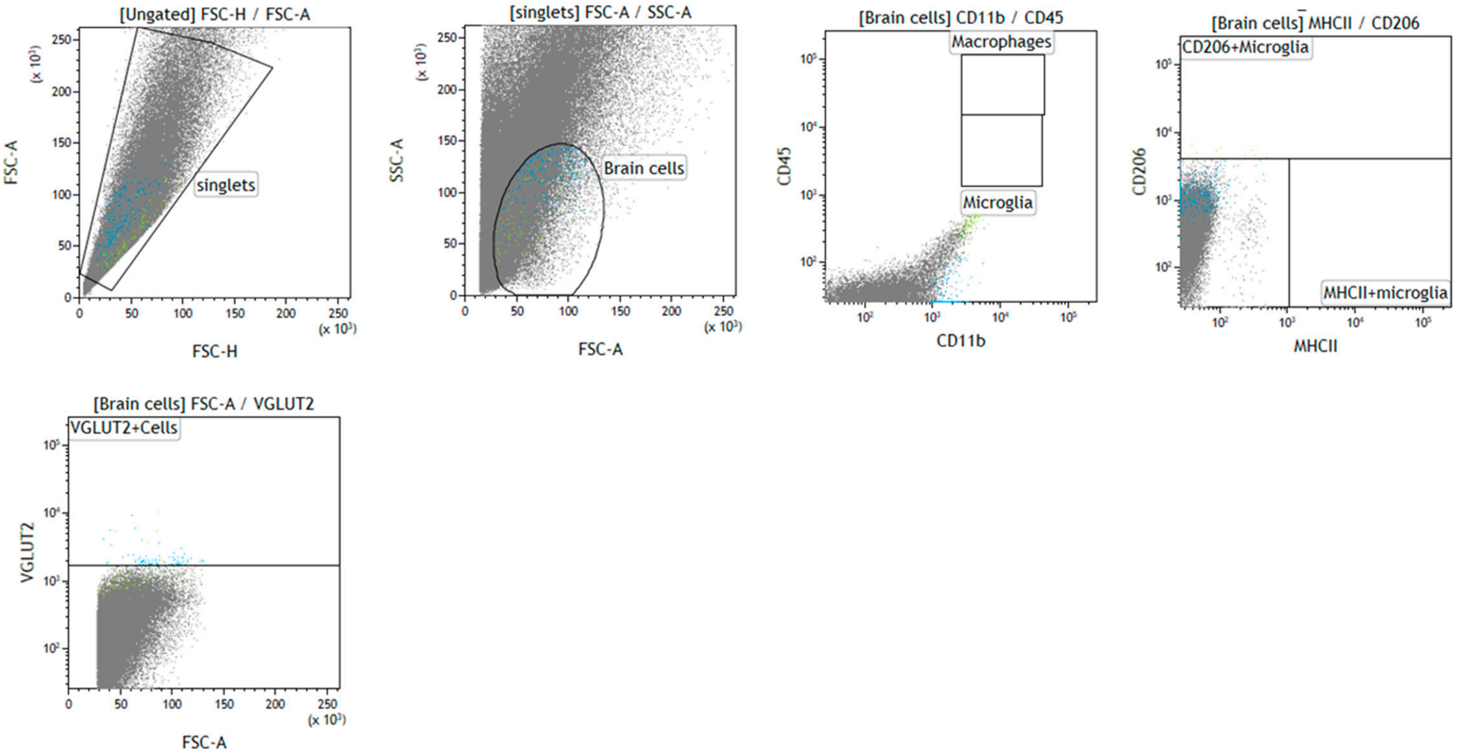

Supplement: Supplementary file 1 [file biology-11-00938-s001.zip › biology-1729232-supplementary.pdf]
